# Ärztinnen in der Orthopädie und Unfallchirurgie in Deutschland: ein aktueller Status quo

**DOI:** 10.1007/s00132-020-04048-7

**Published:** 2020-12-08

**Authors:** Patricia M. Lutz, Julia Lenz, Andrea Achtnich, Stephanie Geyer

**Affiliations:** 1 Abteilung und Poliklinik für Sportorthopädie, Klinikum rechts der Isar, TU München, Ismaningerstr. 22, 81675 München, Deutschland; 2 Zentrum für Orthopädie und Unfallchirurgie, Uniklinikum Marburg, Marburg, Deutschland

**Keywords:** Karriereleiter, Arbeitsplatz, Medizinische Gesellschaften, Mediziner, Frauen, Career ladders, Job site, Medical societies, Physicians, Woman

## Abstract

**Hintergrund:**

Geschlechtsspezifische Unterschiede in der Arbeitswelt allgemein und im Bereich der Orthopädie und Unfallchirurgie im speziellen sind immer noch deutlich erkennbar, geraten aber immer stärker ins gesellschaftliche Bewusstsein.

**Ziel:**

Analyse der geschlechtsspezifischen Entwicklung im Fachbereich Orthopädie und Unfallchirurgie in Deutschland über die vergangenen 15 Jahre und Erhebung des Status quo.

**Methode:**

Erfassung der Entwicklung von Absolventinnen des Humanmedizinstudiums, des Frauenanteils in der vertragsärztlichen und klinischen Versorgung und des geschlechtsspezifischen Erwerbs einer orthopädischen/unfallchirurgischen Zusatzbezeichnung. Auswertung der geschlechtsspezifischen Mitglieder- oder Teilnehmerzahlen der entsprechenden Fachgesellschaften und der größten deutschen Kongresse für Orthopädie und Unfallchirurgie sowie der Habilitationszahlen im Bereich Orthopädie.

**Ergebnisse und Diskussion:**

Der Anteil an Ärztinnen in verschiedenen Bereichen der Orthopädie und Unfallchirurgie in Klinik und Wissenschaft steigt. In Führungspositionen in Kliniken, beim Erwerb von Zusatzbezeichnungen und in den Vorständen von Fachgesellschaften besteht immer noch eine deutliche Diskrepanz. In manchen Bereichen, wie Kinder‑, Hand- oder Fuß‑/Sprunggelenkchirurgie ist der Frauenanteil höher als in anderen Teilbereichen der Orthopädie und Unfallchirurgie.

## Abstract (en)

**Background:**

Gender-specific differences in the workplace in general and in the field of orthopedics and trauma surgery in particular are still clearly identifiable, and are becoming more and more pronounced in the social consciousness.

**Objective:**

Analysis of the gender-specific development in the specialist field of orthopedics and trauma surgery in Germany over the past 15 years and a survey of the status quo.

**Methods:**

Assessment of the development of female graduates in the study of human medicine, the proportion of women in accredited and clinical care and the gender-specific acquisition of an additional orthopedic/trauma surgical title. Evaluation of gender-specific numbers of members or participants in the relevant professional associations and the largest German conferences on orthopedics and trauma surgery, as well as the habilitation figures in the field of orthopedics.

**Results and discussion:**

The proportion of female doctors in various fields of orthopedics and trauma surgery in the clinic and in science is increasing. There is still a clear discrepancy in leading positions in clinics, in the acquisition of an additional title, and on the boards of professional associations. In some fields, such as pediatric and hand/foot/ankle surgery, the proportion of women is higher than in other sub-areas of orthopedics and trauma surgery.

## Hintergrund

Geschlechtsspezifische Unterschiede in der allgemeinen Arbeitswelt bleiben ein häufig gesellschaftlich diskutiertes Thema, wie die Diskussion um den Gender-Pay-Gap kürzlich zeigte [1]. Im Jahr 2018 betrug der Anteil der mit Frauen besetzten Führungspositionen global 24 % [2]. Dieses Ungleichgewicht der Geschlechter ist zuletzt auch in akademischen und beruflichen Bereichen der Medizin immer stärker ins Bewusstsein geraten [3–5]. Trotz einem zunehmend steigenden Anteil an Absolventinnen im Fachgebiet der Humanmedizin in Deutschland (61,3 % Frauen im Jahr 2018) [6], lag der prozentuale Anteil von Ärztinnen in einer Führungsposition an universitären Kliniken in Deutschland im Jahr 2016 bei 10 % [3]. In chirurgischen Fachgebieten scheint dieses Ungleichgewicht am stärksten ausgeprägt zu sein [3]. Weltweite Daten konnten zeigen, dass im Fachbereich Orthopädie und Unfallchirurgie (O&U) bis heute eine starke Differenz vorliegt: Die Zahlen von orthopädisch-unfallchirurgisch tätigen Ärztinnen variieren weltweit von 3 % in England, 5 % in Australien, 5,1 % in Neuseeland, 5,9 % in der Schweiz, 6 % in den USA, 10,7 % in Österreich und 11,2 % in Kanada [4, 7–10]. Im Assistenzarztbereich liegt der Anteil an weiblichen Ärztinnen in englischsprachigen Ländern (14 % in den USA, 19 % in Kanada, 19 % in Neuseeland) etwas höher, ist aber geringer als in anderen chirurgischen Fachgebieten [9, 11–13]. Einige Ursachen für die anhaltenden geschlechtsspezifischen Unterschiede in der O&U wurden in der aktuellen Literatur bereits beschrieben. Hierzu zählen vor allem: wenig Kontakt zum Fach während des Medizinstudiums, das Fehlen von Mentorinnen, Vorurteile hinsichtlich des Geschlechts und Bedenken hinsichtlich der Auswirkungen auf den Lebensstil sowie fehlende Vereinbarkeit von Familie und Beruf [10, 11, 14]. Gedanken wie „Orthopädie und Unfallchirurgie ist nichts für Frauen“ scheinen durch das Fehlen von weiblichen Vorbildern verstärkt zu werden [8]. Der „Glass-ceiling“-Effekt, der eine unsichtbare Grenze für Frauen beschreibt, eine führende Rolle zu erreichen, besteht heutzutage im Fachbereich O&U weiterhin.

Ziel dieser Arbeit ist es, die geschlechtsspezifische Entwicklung im Fachbereich O&U in Deutschland in den letzten 15 Jahren zu analysieren und einen aktuellen Status quo zu erheben. Hierzu erfolgte eine Analyse der Entwicklung von Absolventinnen des Humanmedizinstudiums, des Frauenanteils in der vertragsärztlichen und klinischen Versorgung sowie die geschlechtsspezifische Auswertung des Erwerbs einer orthopädisch/unfallchirurgischen Zusatzbezeichnung. Darüber hinaus wurden im wissenschaftlichen Bereich orthopädische und/oder unfallchirurgische Fachgesellschaften in Deutschland, der größte deutsche Kongress für Orthopädie und Unfallchirurgie (DKOU) und Habilitationszahlen im Fachbereich Orthopädie hinsichtlich der Geschlechterverteilung ausgewertet.

Die Hypothese der vorliegenden Arbeit war, dass die Anzahl von Ärztinnen im Bereich der O&U steigt, sich aber weiterhin ein deutliches Ungleichgewicht in der Geschlechterverteilung darstellt.

## Methoden

### Datenerfassung

Im März und April 2020 wurden im Rahmen dieser Studie von verschiedenen Stellen statistische Daten bezüglich der Geschlechterverteilung in der Orthopädie und Unfallchirurgie in den letzten 15 Jahren in Deutschland eingeholt:

- Statistische Daten der Absolventinnen und Absolventen des Humanmedizinstudiums in Deutschland (Gesundheitsberichterstattung des Bundes)
- Entwicklung in deutschen Kliniken (Informationssystem der Gesundheitsberichterstattung des Bundes, getragen vom Robert Koch-Institut (RKI) und dem Statistischen Bundesamt)
- Entwicklung in der vertragsärztlichen Versorgung im ambulanten Bereich (Bundesarztregister der kassenärztlichen Bundesvereinigung)
- Daten zur Anerkennung von Zusatzweiterbildungen von 2016 bis 2018 im Bereich der Orthopädie und Unfallchirurgie (Bundesärztekammer)
- Geschlechtsanalyse der Leitung einer orthopädischen/unfallchirurgischen Universitätsklinik (Homepage der 37 deutschen Universitätskliniken, an denen das Studium der Humanmedizin staatlich möglich ist)
- Statistische Daten zum Mitgliedsstatus und zur Geschlechterverteilung der Deutschen Gesellschaft für Orthopädie und Unfallchirurgie (DGOU), der Deutschen Gesellschaft für Orthopädie und orthopädische Chirurgie (DGOOC), der Deutschen Gesellschaft für Unfallchirurgie (DGU), der Deutschen Wirbelsäulengesellschaft (DWG), der Deutschen Gesellschaft für Handchirurgie (DGH), der Deutschen Kniegesellschaft (DKG), der Deutschen Assoziation für Fuß und Sprunggelenk (D.A.F.), der Gesellschaft für Arthroskopie und Gelenkchirurgie (AGA) und der Deutschen Vereinigung für Schulter- und Ellenbogenchirurgie (DVSE) wurden von der jeweiligen Gesellschaft erfragt.
- Das Programm des größten unfallchirurgisch-orthopädischen Kongresses DKOU aus dem Jahr 2019 wurde exemplarisch hinsichtlich der Geschlechterverteilung analysiert und nach Art der Präsentation (Expertensitzungen [O&U Basics, O&U Advanced], des Vortrages [O&U Abstract] und Posterpräsentation) und des Vorsitzes der jeweiligen Session ausgewertet. Firmen-Workshops und Kurse/Akademien wurden in der Auswertung nicht berücksichtigt. Die geschlechtsspezifische Statistik von Teilnehmerinnen und Teilnehmern des DKOU wurde von der Kongressorganisation eingeholt.
- Daten von erfolgreich abgeschlossenen Habilitationen in den Jahren 2005 bis 2019 im Fachbereich Orthopädie (Hochschulstatistik des statistischen Bundesamtes)

### Analyse

Die statistische Auswertung wurde mit Excel 2019 (Microsoft, Redmond, WA, USA) vorgenommen. Die Angabe der Ergebnisse erfolgte in absoluten Zahlen und in prozentualen Anteilen. Die Entwicklung der Zahlen wurde in entsprechenden Diagrammen bzw. in prozentualen Berechnungen wiedergegeben.

## Ergebnisse

### Absolventen im Bereich der Humanmedizin

Der Anteil an Frauen, die in Deutschland das Studium der Humanmedizin erfolgreich absolvierten, stieg von 54,3 % im Jahr 2005 auf 62,7 % im Jahr 2010 und ist im Jahr 2018 leicht auf 61,3 % gefallen [6, 15].

### Vertragsärztliche Versorgung

Der Anteil an Orthopädinnen in der vertragsärztlichen Versorgung in Deutschland ist von 2009 bis 2018 von 10,2 auf 12,3 % gestiegen [16].

### Klinikversorgung

**Figure 1:**
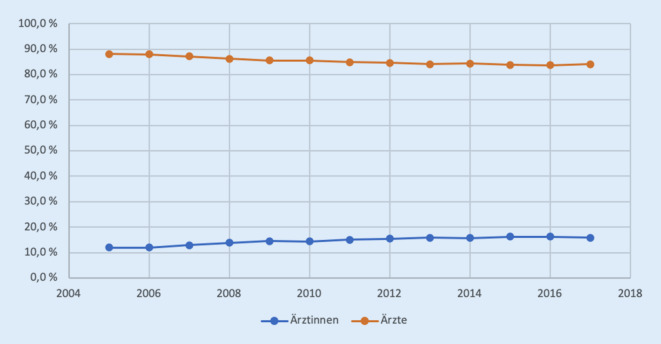

Die Entwicklung hinsichtlich der Geschlechtsverteilung von Ärztinnen und Ärzten in der Orthopädie in allgemeinen Krankenhäusern in Deutschland ist in Abb. 1 dargestellt.

**Figure 2:**
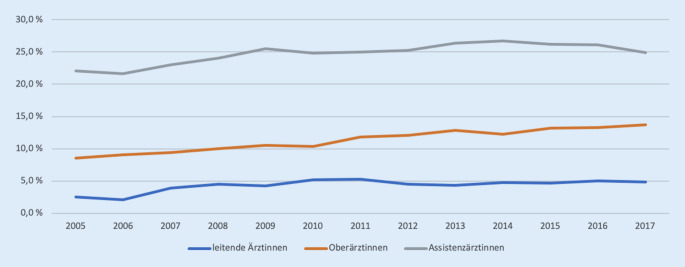

Abb. 2 zeigt die Entwicklung von Ärztinnen abhängig von dem jeweiligen Berufsstand in der Orthopädie in deutschen Kliniken von 2005 bis 2017. Insgesamt konnte eine Zunahme der Ärztinnen in der Orthopädie gezeigt werden. Prozentual gesehen ist eine Steigerung um 12,9 % bei den Assistenzärztinnen, um 60,1 % bei den Oberärztinnen und um 94,0 % bei den leitenden Ärztinnen zu verzeichnen.

### Leitung der Orthopädie/Unfallchirurgie an deutschen Universitäten

Im März 2020 sind an 37 deutschen Universitätskliniken insgesamt drei Frauen (5,1 %) in der leitenden Rolle als Direktorin tätig. Zwei Direktorinnen leiten dabei eine rein orthopädische Klinik. Insgesamt leiten 59 Direktoren/Direktorinnen die Abteilungen für Orthopädie oder/und Unfallchirurgie an deutschen Universitätskliniken.

### Zusatzbezeichnung Unfallchirurgie/Orthopädie

In Tab. 1 ist der Erwerb einer Zusatzbezeichnung im Fach Orthopädie und Unfallchirurgie, unterteilt nach Geschlecht, aus den Jahren 2016 bis 2018 ersichtlich. Die Voraussetzung für den Erwerb einer solchen Zusatzbezeichnung ist die Facharztanerkennung Orthopädie und Unfallchirurgie.

**Table 1:** 

	2016	2017	2018
Handchirurgie			
w (*N*, %)	25 (24,8 %)	30 (28,0 %)	27 (24,3 %)
m (*N*, %)	76 (75,2 %)	77 (72,0 %)	84 (75,7 %)
Kinderorthopädie			
w (*N*, %)	12 (32,4 %)	11 (31,4 %)	11 (24,4 %)
m (*N*, %)	25 (67,6 %)	24 (68,6 %)	34 (75,6 %)
Orthopädische Rheumatologie			
w (*N*, %)	0 (0 %)	2 (18,2 %)	0 (0 %)
m (*N*, %)	7 (100 %)	9 (81,8 %)	3 (100 %)
Spezielle orthopädische Chirurgie			
w (*N*, %)	4 (4,6 %)	5 (6,4 %)	4 (4,6 %)
m (*N*, %)	83 (95,4 %)	73 (93,6 %)	83 (95,4 %)
Spezielle Unfallchirurgie			
w (*N*, %)	39 (16,3 %)	17 (8,0 %)	26 (11,6 %)
m (*N*, %)	201 (83,7 %)	196 (92,0 %)	199 (88,4 %)

Anerkennung von Zusatzweiterbildungen im Bereich Orthopädie/Unfallchirurgie der Bundesärztekammer Deutschland in den Jahren 2016 bis 2018, aufgeteilt nach Geschlecht [18]

*w* weiblich, *m* männlich

### Gesellschaften

Aktuelle Mitgliederzahlen, die absolute prozentuale Entwicklung des Frauenanteils und die Geschlechtsverteilung im Vorstand der jeweiligen Gesellschaft sind in Tab. 2 zusammengefasst. Im Jahr 2020 sind alle Präsidenten der aufgeführten Gesellschaften männlich.

**Table 2:** 

Gesellschaft	Mitglieder (% weiblich)	Entwicklung in %	Vorstand (m : w)
DGOU	27,2	+1,0 seit 2015	13:0
DGOOC	13,7	+0,3 seit 2015	8:0
DGU	12,1	−0,2 seit 2015	9:0
DWG	9,6	+4,6 seit 2011	14:1
DGH	25,6	+5,6 seit 2015	35:7
DKG	6,2	/	17:1
D.A.F	26,4	/	4:2
AGA	13	+2,6 seit 2015	12:0
DVSE	12,5	+1,9 seit 2015	5:0

Weibliche Mitglieder in %, Entwicklung des Anteils an weiblichen Mitgliedern in %, Aufteilung des Vorstands (männlich : weiblich)

DGOU Deutsche Gesellschaft für Orthopädie und Unfallchirurgie, DGOOC Deutsche Gesellschaft für Orthopädie und orthopädische Chirurgie, DGU Deutsche Gesellschaft für Unfallchirurgie, DWG Deutsche Wirbelsäulengesellschaft, DGH Deutsche Gesellschaft für Handchirurgie, DKG Deutsche Knie Gesellschaft, D.A.F. Deutsche Assoziation für Fuß und Sprunggelenk, AGA Gesellschaft für Arthroskopie und Gelenkchirurgie, DVSE Deutsche Vereinigung für Schulter- und Ellenbogenchirurgie

Im studentischen Bereich sind die Mitgliederzahlen hinsichtlich des Geschlechtes in der DGOU und der DVSE seit 2015 ausgeglichen, in der DGH gibt es seit 2018 mehr weibliche studentische Mitglieder als männliche. Im Assistenzarztbereich der DGH liegt der Anteil an weiblichen Mitgliedern aktuell bei 57,1 %.

### DKOU

Der Anteil an Teilnehmerinnen beim DKOU ist von 2010 bis 2019 von 19 auf 21 % gestiegen. Dabei wurde der Berufsstand der Teilnehmerinnen nicht erfasst.

**Figure 3:**
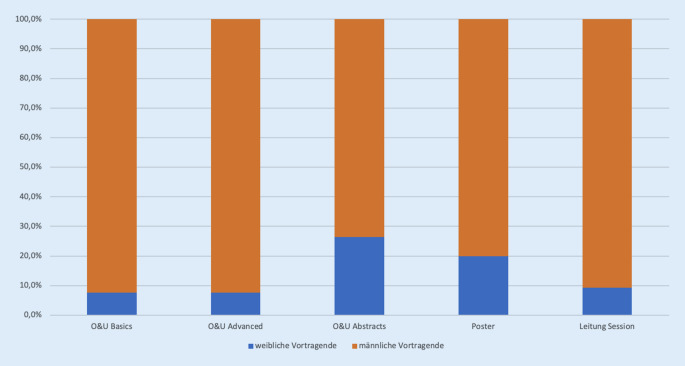

Vorsitzende waren zu 90,7 % männlich. Geladene Expertinnen und Experten, die einen Vortrag im Bereich O&U Basic und O&U Experten hielten, waren zu 92,3 % männlich. Vorträge, die nach positivem Votum zu einem wissenschaftlichen Abstract erfolgten, wurden in 26,4 % von Frauen präsentiert. Im Bereich der Posterpräsentationen lag der Anteil von präsentierenden Frauen bei 20 %. Abb. 3 zeigt die Verteilung von männlichen und weiblichen Vortragenden und der Leitung einer Sitzung auf dem DKOU-Kongress 2019. Die drei Kongresspräsidenten waren männlich.

### Habilitationen

**Figure 4:**
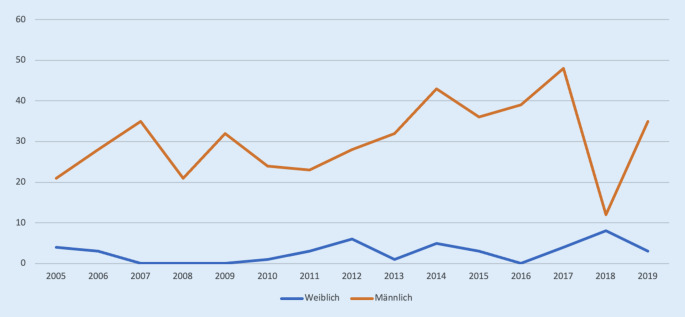

Zwischen den Jahren 2005 und 2019 haben 41 (8,2 %) Frauen und 457 (91,8 %) Männer eine Habilitation im Fachbereich Orthopädie an einer deutschen Universität erfolgreich abgeschlossen (Abb. 4).

## Diskussion

In der vorliegenden Arbeit konnte gezeigt werden, dass der Anteil an Ärztinnen im Fachbereich Orthopädie und Unfallchirurgie in Deutschland in den letzten Jahren zugenommen hat. Der Frauenanteil in Führungspositionen hat sich seit dem Jahr 2005 verdoppelt und lag im Jahr 2017 bei 4,8 %. 6–27 % der Mitglieder in den einzelnen Fachgesellschaften sind weiblich. In diesem Bereich konnte in den letzten Jahren ein leichter Anstieg verzeichnet werden. Demgegenüber zeigt sich im Bereich der studentischen Mitglieder das Verhältnis von Frauen zu Männern seit Jahren als ausgeglichen. Im Vorstand der entsprechenden Gesellschaften liegt weiterhin eine seltene Vertretung des weiblichen Geschlechts vor.

Auch im akademischen Bereich (DKOU 2019 [19], Habilitationen) konnte ein deutliches Ungleichgewicht der Geschlechter dargestellt werden.

Trotz einer stetig steigenden Anzahl an Hochschulabsolventinnen stellen Ärztinnen im Fachbereich O&U in Deutschland nach wie vor eine Minderheit dar. Auch in anderen Ländern liegen entsprechende Daten vor, die das bestätigen [4, 7–9]. Mögliche Hürden auf dem Weg zur orthopädisch-unfallchirurgischen Chirurgin und einer akademischen Karriere wurden in verschiedenen Veröffentlichungen wie folgt beschrieben: fehlender Kontakt zu dem Fachgebiet O&U und muskuloskelettalen Themen während des Studiums [11, 13, 14, 21, 22], negativer Bias gegenüber Frauen [5] und das Fehlen von weiblichen Vorbildern [5, 13, 23]. Es konnte bereits gezeigt werden, dass ein früher Kontakt mit dem Fachgebiet O&U während des Studiums dazu führt, dass mehr weibliche Ärztinnen dieses Fachgebiet als Spezialisierung wählen [21, 23]. Weiterhin scheint jedoch die Annahme zu kursieren, dass eine hohe körperliche Kraft für die Durchführung von operativen Eingriffen in der O&U erforderlich sei [8]. Eine ungünstige Work-Life-Balance und eine schlechte Vereinbarkeit von Familie und Beruf könnten laut der aktuellen Literatur weitere Gründe für die Minderheit der Frauen in diesem Bereich sein [8, 24]. Generell lassen die Ergebnisse dieser Studie aber vermuten, dass das Interesse bei Studentinnen für das Fachgebiet mindestens so ausgeprägt ist wie bei Studenten. Die Anzahl der Assistenzärztinnen im Fachbereich O&U ist in den letzten Jahren stetig gestiegen und mit der Anzahl an Assistenzärztinnen in Kanada vergleichbar [8]. Trotzdem finden sich in Führungspositionen nach wie vor nur wenig Frauen. Erklären lässt sich dies am ehesten dadurch, dass Frauen in der Regel am Ende der Assistenzarztzeit oder mit Erwerb des Facharzttitels Kinder bekommen. Genau dieser Zeitraum gilt jedoch als wegweisend für eine Karriere, sei es auf klinischer Ebene (Erwerb einer Zusatzbezeichnung, Aufstieg zur Oberärztin), auf wissenschaftlicher Ebene (Habilitation, aktive Kongressgestaltung) oder in Fachgesellschaften (Positionen in Komitees oder Vorständen). Nach einem Wiedereinstieg arbeiten Ärztinnen oft in Teilzeit, was sich häufig nur schwer mit dem weiteren Ausbau der eigenen Karriere vereinbaren lässt [25]. Der Anteil an Frauen in Führungspositionen steigt zwar an, ein ausgeglichenes Verhältnis von Ärzten und Ärztinnen in Führungspositionen in Deutschland im Fachbereich O&U ist aber nicht erreicht. Mögliche Hindernisse neben der Familienplanung könnten laut der Literatur ein traditionelles Rollenbild, männlich dominierte hierarchische Strukturen in den Kliniken und ein fehlendes Netzwerk sein. Als weitere Ursachen werden eine unzureichende Karriereplanung und eine Fehleinschätzung der Karrieremöglichkeiten durch Ärztinnen aufgeführt [25]. Da speziell eine Karriere an einer Universität abhängig von Forschungstätigkeiten ist, spielen auch Hürden im Bereich der medizinischen Wissenschaft eine Rolle: Artikel werden seltener publiziert [26], Forscherinnen werden teilweise schlechter bezahlt [27], erhalten weniger Fördergelder [28] und seltener Auszeichnungen [29]. Derzeit gibt es einige gute Ansätze zur Verbesserung dieser Defizite. Dazu zählen zum Beispiel Mentoring-Programme, spezielle Förderprogramme im Wissenschaftsbereich und die Gründung von Netzwerken, wie zum Beispiel „Die Orthopädinnen e. V.“ und der „Deutsche Ärztinnenbund e. V.“. Ärztinnen bekommen dadurch die Möglichkeit, die eigene Karriere voranzutreiben. Ein weiterer wichtiger Punkt, um die Geschlechterunterschiede im akademischen Bereich zu minimieren, stellt die Verbesserung des Arbeitsumfeldes dar. So zeigten Umfragen, dass 76 % aller Frauen und 62 % der Männer von Diskriminierung am Arbeitsplatz im Bereich der akademischen Medizin in Deutschland berichten [30].

In den Bereichen Kinderorthopädie, Handchirurgie und Fuß‑/Sprunggelenkchirurgie ist der Anteil an Ärztinnen höher als in den anderen Subspezialisierungen. Dies legt nahe, dass diese Subspezialisierungen attraktiver für Frauen zu sein scheinen. Ursächlich hierfür könnte die Annahme sein, dass unter anderem die Vereinbarkeit von Beruf und Familie in einem Arbeitsumfeld mit guten Optionen zur elektiven, operativen Tätigkeit besser gelingt.

Die Analyse konnte zeigen, dass auf dem DKOU 2019 bis zu 26,4 % der wissenschaftlichen Vorträge durch Ärztinnen oder Wissenschaftlerinnen präsentiert wurden. Dies weist auf ein reges Interesse und eine gute Teilnehmerinnenquote hin, wenn man die generellen Zahlen von Ärztinnen in dem Fachbereich betrachtet.

Im internationalen Vergleich wurden allerdings nur wenige Spezialistinnen auf dem Gebiet der O&U eingeladen, um einen Expertenvortrag zu halten [8]. Gründe hierfür könnten zum einen der geringere Anteil an Frauen im akademischen Bereich, zum anderen die schon existierende Theorie sein, dass Männer zu einem größeren Teil Männer förderten [31]. Auch Vorsitzende auf dem DKOU waren überwiegend männlich. Eine Untersuchung aus dem Fachbereich der Mikrobiologie hat gezeigt, dass gemischte Vorsitze auf Kongressen dazu führen, dass sowohl im Auditorium als auch im Bereich der Vortragenden eine verbesserte Geschlechtsbalance erreicht werden kann [32]. Grundsätzlich besteht hier eine weitere Möglichkeit der Verringerung der geschlechterspezifischen Unterschiede.

Basierend auf den Daten dieser Studie kann festgestellt werden, dass der Anteil an Frauen im Assistenzarztbereich zunimmt, jedoch in höheren Positionen wieder abnimmt. Bereits bestehende Netzwerke und Förderprogramme sollten weiter ausgebaut und eine Inanspruchnahme ermöglicht werden. Das Ziel sollte die Annäherung an ein ausgeglichenes Geschlechterverhältnis im Fachbereich O&U sein. Nachweislich bietet Diversität die Möglichkeit innovativer Ansätze, qualitativ besserer Entscheidungen, erhöhter Produktivität und von lösungsorientierterem Handeln [33]. Speziell im Gesundheitsbereich konnte außerdem gezeigt werden, dass eine ausgeglichene Geschlechtsverteilung zu einer hochwertigeren Patientenversorgung sowie zu einem besseren Verständnis von zu behandelnden Patientinnen und Patienten führen kann [8]. Die vorliegende Arbeit soll als Grundlage für weitere Untersuchungen dienen.

Als Limitation dieser Studie kann genannt werden, dass Personen, die sich nicht als Frau oder Mann identifizieren, in dieser Studie nicht berücksichtigt wurden, da keine Daten zur Verfügung standen. Vergleichbare Daten aus anderen Ländern sind mangelhaft und nur aus Kanada und Neuseeland aktuell. Da es sich um eine deskriptive Datenanalyse handelte und die Daten von externen Organisationen zur Verfügung gestellt wurden, kann als weitere Limitation genannt werden, dass nicht alle Daten auf dem aktuellen Stand (2020) präsentiert werden konnten. Retrospektiv konnten wir, bezogen auf den DKOU 2019, nicht feststellen, wie hoch der Anteil an Frauen war, die einen Abstract für den Kongress eingereicht haben und ob hier ein Selektionsbias vorlag.

## Fazit für die Praxis

- Aktuell steigt der Anteil an Ärztinnen auf verschiedenen Ebenen im Fachbereich O&U (Orthopädie und Unfallchirurgie) sowohl in der Klinik als auch in der Wissenschaft, ein ausgeglichenes Geschlechterverhältnis ist in Deutschland jedoch noch keine Realität.
- Obwohl über 60 % der Medizinabsolventinnen und Absolventen in Deutschland und 25 % aller Assistenzärztinnen und Assistenzärzte in der O&U weiblich sind, besteht über die Facharztanerkennung hinaus in allen Bereichen, die zu einer medizinischen Karriere gezählt werden (Position in Klinik, Erwerb einer Zusatzbezeichnung, Vorstand von Fachgesellschaften und Wissenschaft), eine deutliche geschlechterspezifische Diskrepanz.
- In den Bereichen Kinderorthopädie, Handchirurgie und Fuß‑/Sprunggelenkchirurgie ist der Anteil an Chirurginnen höher als in anderen Teilbereichen der O&U.
- Auf dem DKOU (Deutsche Gesellschaft für Orthopädie und Unfallchirurgie) 2019 waren Vortragende im Bereich der Expertenvorträge und im Vorsitz von Sitzungen überwiegend männlich. 26,4 % der wissenschaftlichen Vorträge wurden von Frauen präsentiert.
- Ärztinnen und Wissenschaftlerinnen im Fachbereich O&U können von Fördermöglichkeiten und Netzwerken profitieren. Diese Möglichkeiten mit Aussicht auf eine akademische Karriere sollten als Chance wahrgenommen werden.

## Abkürzungen

AGA: Gesellschaft für Arthroskopie und Gelenkchirurgie
D.A.F.: Deutsche Assoziation für Fuß und Sprunggelenk
DGH: Deutsche Gesellschaft für Handchirurgie
DGOOC: Deutsche Gesellschaft für Orthopädie und orthopädische Chirurgie
DGOU: Deutsche Gesellschaft für Orthopädie und Unfallchirurgie
DGU: Deutsche Gesellschaft für Unfallchirurgie
DKG: Deutsche Kniegesellschaft
DKOU: Deutscher Kongress für Orthopädie und Unfallchirurgie
DVSE: Deutsche Vereinigung für Schulter- und Ellenbogenchirurgie
DWG: Deutsche Wirbelsäulengesellschaft
O&U: Orthopädie und Unfallchirurgie
RKI: Robert Koch-Institut
